# Einflussfaktoren und Ergebnisse von Konversionen in der minimal-invasiven Leberchirurgie: Eine Zentrumsanalyse von mehr als 1200 konsekutiven Fällen

**DOI:** 10.1007/s00104-025-02374-0

**Published:** 2025-09-11

**Authors:** Nils Haep, Lucas Illies, Yun Jung Choi, Sebastian Knitter, Philipp Haber, Felix Krenzien, Nathanael Raschzok, Johann Pratschke, Wenzel Schöning

**Affiliations:** 1 Klinik für Chirurgie, Campus Charité Mitte , Campus Virchow-Klinikum, Charité – Universitätsmedizin Berlin, Gliedkörperschaft der Freien Universität Berlin und der Humboldt-Universität zu Berlin, Augustenburger Platz 1, 13353 Berlin, Deutschland; 2 Clinician Scientist Programm, Berliner Institut für Gesundheit der Charité (BIH), Anna-Louisa-Karsch-Str. 2, Berlin, Deutschland

**Keywords:** Minimal-invasive Leberchirurgie, Konversionsrate, Konversionsgründe, Robotergestützte Chirurgie, Risikofaktoren, Minimally invasive liver surgery, Conversion rate, Reason for conversion, Robotic surgery, Risk factors

## Abstract

**Hintergrund:**

Die minimal-invasive Leberchirurgie ist mit niedrigeren Komplikationsraten im Vergleich zur offenen Leberchirurgie assoziiert. Aufgrund der wachsenden Akzeptanz und zunehmenden Erfahrung mit minimal-invasiven Leberresektionen sind Konversionen auf ein offenes Verfahren zwar selten geworden, gehen jedoch mit einer Verschlechterung des postoperativen Outcomes einher.

**Zielsetzungen:**

Unser Ziel war es, die Risikofaktoren für eine Konversion und die postoperativen Ergebnisse von Patienten bei minimal-invasiven Leberresektionen zu untersuchen.

**Materialien und Methoden:**

Wir führten eine Post-hoc-Analyse von 1209 zwischen 2015 bis 2024 an unserem Zentrum durchgeführten konsekutiven minimal-invasiven Leberresektionen durch. Risikofaktoren und perioperative Ergebnisse der Konversion zu offenen Verfahren wurden analysiert.

**Ergebnisse:**

Die Konversionsrate betrug 4,1 % und die Hemihepatektomie links war mit einer erhöhten Konversionsrate assoziiert. Die Parenchymdissektoren Harmonic Ace® und Waterjet® waren mit einer reduzierten Konversionsrate assoziiert. Die häufigsten Gründe für eine Konversion waren technische Gründe und Verwachsungen. Major-Komplikationen (Clavien Dindo ≥ III) innerhalb der ersten 90 Tage traten in 18,3 % der Fälle auf und die Konversionen waren mit einer erhöhten Komplikationsrate assoziiert.

**Schlussfolgerung:**

Zusammenfassend hebt diese Studie die Einflussfaktoren hervor, die mit einer offenen Konversion bei minimal-invasiven Leberresektionen korrelieren. Die offene Konversion war ein unabhängiger Prädiktor für eine erhöhte Komplikationsrate.

**Graphic abstract:**

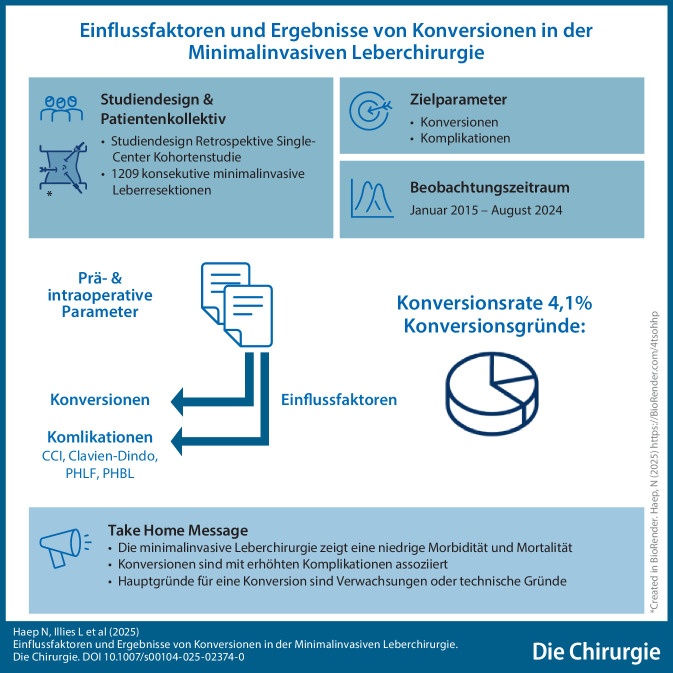

**Zusatzmaterial online:**

Die Online-Version dieses Beitrags (10.1007/s00104-025-02374-0) enthält zusätzliche Tabellen.

## Abstract (en)

**Background:**

Minimally invasive liver surgery is associated with lower complication rates compared to open liver surgery. Due to the growing acceptance and increasing experience with minimally invasive liver resections, open conversions have become rare but are associated with inferior postoperative outcomes.

**Objectives:**

We aimed to investigate the risk factors for conversion and outcomes in patients undergoing minimally invasive liver resections.

**Material and methods:**

We performed a post hoc analysis of 1209 consecutive minimally invasive liver resections at our center between 2015 and 2024. The factors and perioperative outcomes of conversion to open procedures were analyzed.

**Results:**

The conversion rate was 4.1% and left hemihepatectomy was associated with an increased conversion rate. The Harmonic Ace® and Waterjet® parenchyma dissectors were associated with lower conversion rates. The most common reasons for conversion were technical and adhesions. Major complications (Clavien Dindo ≥ III) within the first 90 days occurred in 18.3% of cases and conversions were associated with an increased complication rate.

**Conclusion:**

In conclusion, this study highlights the influencing factors which correlate to open conversion in minimally invasive liver resections. Open conversion was an independent predictor of an increased complication rate.

## Hintergrund

Die minimal-invasive Leberchirurgie hat sich in den letzten Jahrzehnten zunehmend als Standardverfahren für resezierbare Lebertumoren etabliert [10–12, 17, 19]. Neben geringeren postoperativen Schmerzen, kürzeren Krankenhausaufenthalten und einer kürzeren Zeit bis zur adjuvanten Chemotherapie, bietet die minimal-invasive Leberchirurgie auch eine niedrigere Morbidität im Vergleich zur offenen Chirurgie [1, 4–6, 8, 24]. Trotz dieser Vorteile stellt die Konversion einer minimal-invasiven zu einer offenen Operation ein relevantes klinisches Ereignis dar. Konversionen können mit einer erhöhten postoperativen Morbidität assoziiert sein, was die Notwendigkeit einer präzisen Risikoabschätzung und -vermeidung unterstreicht.

## Fragestellung

Ziel dieser Studie war es, anhand von über 1200 konsekutiven Fällen unseres Zentrums die Risikofaktoren für eine Konversion sowie deren Einfluss auf das perioperative Outcome systematisch zu analysieren.

## Material und Methoden

### Studiendesign und Untersuchungsmethoden

Eingeschlossen wurden alle Patienten, die sich zwischen Januar 2015 und August 2024 in der Klinik für Chirurgie, Campus Charité Mitte und Campus Virchow-Klinikum, Charité-Universitätsmedizin Berlin, einer minimal-invasiven Leberresektion unterzogen haben. Alle eingeschlossenen Patienten stimmten der Erhebung und Verwendung ihrer persönlichen und medizinischen Daten für Forschungszwecke zu. Alle Daten wurden in Übereinstimmung mit der Allgemeinen Datenschutzverordnung und den lokalen Datenschutzgesetzen erhoben, gespeichert und verarbeitet. Die Studie wurde in Übereinstimmung mit den ethischen Standards der Deklaration von Helsinki aus dem Jahr 1975 durchgeführt. Die Ethikkommission der Charité genehmigte die Studie (EA4/084/17).

### Patientenauswahl

Ohne spezifische Indikationen für eine offene Resektion wurde bei Leberresektionen die minimal-invasive Chirurgie bevorzugt. Die Entscheidung für einen laparoskopischen oder robotergestützten Ansatz basierte nicht auf einer Reihe vorgegebener Auswahlkriterien. Bei individueller Indikation wurde vor einer Major-Resektion eine Leberhypertrophieinduktion veranlasst.

### Chirurgische Techniken

**Abb. 1:**
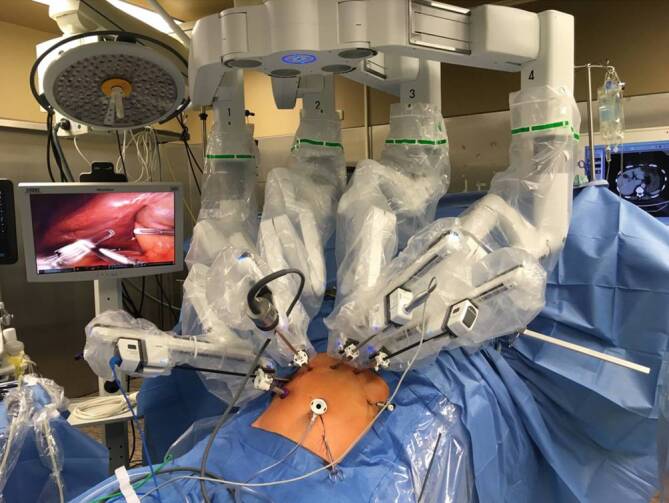
Bild aus dem Operationssaal mit dem DaVinci-Intuitive-Xi-System, das für die robotischen Resektionen verwendet wurde

Für die robotergestützte Chirurgie wurde das da Vinci-Xi-System (Intuitive, Sunnyvale, CA, USA) verwendet (Abb. 1). Die Patienten wurden in die umgekehrte Trendelenburg-Lage gebracht. Vier 8‑mm-Robotertrokare wurden von zwei 12-mm-Hilfstrokaren begleitet ([20]; Abb. 1). Bei der Hybridresektion konnte durch eine minimalinvasive Mobilisation der Leber auf eine quere Oberbauchlaparotomie verzichtet werden. Intraoperativ fand routinemäßig eine Ultraschalluntersuchung statt, um die genaue Lage des Tumors, seine Grenzen, die Nähe zu Gefäß- und Gallengangsstrukturen zu visualisieren und zusätzliche intrahepatische Läsionen auszuschließen. Vor der Parenchymresektion wurde zur Vorbereitung eines intermittierenden Pringle-Manövers das Ligamentum hepatoduodenale isoliert und umschlungen. Es wurden 250 mg Methylprednisolon intravenös vor der Parenchymdissektion verabreicht. Das Pringle-Manöver wurde in 15-minütigen Intervallen durchgeführt, die von 5‑minütigen Reperfusionsperioden getrennt waren. Unabhängig von der Zugangstechnik wurden beim cholangiozellulären Karzinom routinemäßig eine Lymphknotendissektion am Hilus und eine intraoperative Schnellschnittuntersuchung durchgeführt.

Für die Parenchymdissektionen während der Laparoskopie wurden Energiescheren (Harmonic Ace®, Ethicon Inc., Somerville, NJ, USA) und das Thunderbeat® (Olympus K. K., Tokio, Japan) verwendet. Für die tiefere Parenchymdissektion standen zusätzlich der laparoskopische Cavitron-Ultraschallaspirator (CUSA, Valleylab Boulder, CO, USA) und der Wasserstrahl (Waterjet®, Elektromedizin GmbH, Tübingen, Deutschland) zur Verfügung. Bei Verwendung des DaVinci-Xi-Chirurgiesystems wurde die Parenchymdurchtrennung mit einer modifizierten Clamp-Crush-Technik unter Verwendung der gebogenen Harmonic Ace®-Schere durchgeführt. Große Gefäße wurden bei der laparoskopischen und robotergestützten Leberchirurgie entweder geklippt oder mit Klammerstaplern (Echelon®, Ethicon, Somerville, NJ, USA) durchtrennt (Abb. 1).

### Scoring-Systeme

Die Morbidität der Patienten wurde auf Basis des Charlson Comorbidity Index (CCI) durch 19 Grunderkrankungen erfasst. Das perioperative Risiko wurde mithilfe des ASA-Scores bestimmt. Eine Major-Resektion wurde definiert als die Entfernung von mehr als drei Segmenten [22]. Für jede Operation wurde der Schwierigkeitsscore nach IWATE ermittelt, der sich aus einer Punktzahl errechnet, die auf der Lage des Tumors, der Tumorgröße, dem Ausmaß der Leberresektion, der Leberfunktion, der Nähe zu den großen Gefäßen und der Verwendung eines hybriden Ansatzes/einer handassistierten laparoskopischen Leberresektion basiert [13, 15, 23]. Die Schwierigkeitsgrade wurden in „Leicht“ (1–3), „Mittel“ (4–6), „Fortgeschritten“ (7–9) und „Experte“ (10–12) eingeteilt. Die postoperativen Komplikationen, die innerhalb von 90 Tagen nach dem Eingriff auftraten, wurden nach Clavien-Dindo klassifiziert [7]. Die kumulativen Komplikationen wurden auf Basis der Clavien-Dindo-Klassifikation im Comprehensive Complication Index zusammengefasst [21].

### Statistische Analyse

Die Datenanalyse wurde in R und R Studio (Version 4.4.2; Posit PBC, Boston, USA) durchgeführt. Normal verteilte numerische Variablen werden als Mittelwert und Standardabweichung angegeben, nichtnormal verteilte numerische Variablen werden durch den Median zusammen mit dem Interquartilsbereich dargestellt. Für kontinuierliche Variablen wurden lineare uni- und multivariante Regressionsmodelle und für kategorische Variablen logistische Regressionsmodelle verwendet. Monotone Trends wurden mit dem Jonckheere-Terpstra-Test oder dem Cochran-Armitage-Trendtest untersucht. Ein *p*-Wert von ≤ 0,05 wurde als statistisch signifikant angesehen.

## Ergebnisse

Im untersuchten Zeitraum wurden 1209 konsekutive minimal-invasive Leberresektionen durchgeführt, das mediane Alter der Patienten betrug hierbei 62 Jahre, wobei Männer mit 57 % überrepräsentiert waren. Der Großteil der Patienten hatte einen ASA-Score von 3 (52 %), einen medianen Charlson-Komorbiditätsindex von 6 und einen BMI von 25,8 (Tab. 1). Die Indikationen zur Leberresektion waren mit absteigender Häufigkeit kolorektale Lebermetastasen (CRLM) (39 %), hepatozelluläre Karzinome (HCC) (22 %), cholangiozelluläre Karzinome (9,6 %), gutartige Lebertumoren (19 %) und andere maligne Lebertumoren (10 %) (Tab. 1). 41 % der Patienten erhielten eine neoadjuvante Chemotherapie (Tab. 1).

**Tab. 1:** Charakterisierung der präoperativen Parameter in der Gesamtkohorte

Merkmale	*N* = 1209^1^
*Alter*	62 (52–71)
*Geschlecht*	
Weiblich	498 (43 %)
Männlich	659 (57 %)
*Body-Mass-Index*	25,8 (22,7–29,2)
*ASA-Risikoklassifikation*	
ASA 1	55 (4,8 %)
ASA 2	487 (42 %)
ASA 3	599 (52 %)
ASA 4	14 (1,2 %)
*Charlson-Komorbiditätsindex*	6,0 (3,0–8,0)
*Indikation*	
CRLM	470 (39 %)
HCC	265 (22 %)
Benigne Tumoren	227 (19 %)
Andere maligne Tumoren	131 (11 %)
Cholangiokarzinom	116 (9,6 %)
*Neoadjuvante Chemotherapie*	428 (41 %)
*Vorherige Leberoperation*	159 (14 %)
*Maximale Tumorgröße (cm)*	3,5 (2,0–6,0)
*Lokalisation*	
Unilobär	534 (44 %)
Bilobär	675 (56 %)
*Nähe zu großen Gefäßen*	475 (41 %)
*Steatose >* *5* *%*	526 (53 %)
*Steatose (%)*	5 (0–20)
*Fibrose*	
Keine Fibrose	433 (49 %)
F1	263 (30 %)
F2	116 (13 %)
F3	75 (8,5 %)
*Zirrhose*	182 (16 %)
*IWATE-Score*	7,50 (5,00–10,00)
*N*/A	56 (4,6 %)
Leicht	115 (9,5 %)
Mittel	373 (31 %)
Fortgeschritten	338 (28 %)
Experte	327 (27 %)

^1^ Kategorische Variablen werden als Häufigkeiten mit Prozentsätzen dargestellt (z. B. *n* [%]), nicht normalverteilte numerische Variablen werden durch den Median zusammen mit dem Interquartilsbereich dargestellt (z. B. Median [25.–75. Perzentil])

*ASA* American Society of Anaesthesiologists Klassifikation, *CRLM* Kolorektale Lebermetastasen, *HCC* Hepatozelluläres Karzinom

Bei 14 % der Patienten handelte es sich um eine Rezidivleberoperation. Der mediane Durchmesser des größten Tumors betrug 3,5 cm und in 56 % der Fälle handelte es sich um bilobäre Tumoren, wobei 41 % der Tumoren einen engen Lagebezug zu den Gefäßen hatten. Zum Zeitpunkt der Operation lagen in 53 % der Fälle eine Lebersteatose und in 16 % der Fälle eine Leberzirrhose vor (Tab. 1). Der Schwierigkeitsgrad der Operation wurde nach IWATE beurteilt und war wie folgt verteilt: Leicht (14,1 %), Mittel (31 %), Fortgeschritten (28 %) und Experte (27 %; Tab. 1).

Insgesamt wurden 73 % der Eingriffe laparoskopisch, 26 % robotisch und 0,7 % als Hybridresektion durchgeführt (Tab. 2). Davon waren 9,5 % Wedge-Resektionen, 7,6 % multiple Wedge-Resektionen, 14 % Segmentresektionen und 20 % Bisegmentresektionen, 21 % Hemihepatektomien der rechten Seite und 10 % Hemihepatektomien der linken Seite. In 8,4 % der Fälle fand ein kombinierter Leber- und Kolorektaleingriff und in 11 % der Fälle fand eine zweizeitige Leberresektion statt (Tab. 2). In Absteigender Reihenfolge wurden zur Parenchymdissektion das Harmonic Ace® (67 %), Waterjet® (17 %), Thunderbeat® (9,6 %) und andere Parenchymdissektoren (10,5 %) verwendet. Das Pringle-Manöver wurde in 49 % der Fälle mit einem Median von 28 und einem Interquartilsbereich von 15–45 min angewendet. Die Rate von Bluttransfusionen betrug 6,1 % (Tab. 2).

**Tab. 2:** Charakterisierung der intraoperativen Parameter der Gesamtkohorte

Merkmale Operation	*N* = 1209^1^
*Zugang*	
Laparoskopisch	888 (73 %)
Robotisch	312 (26 %)
Hybrid	9 (0,7 %)
*Minor-Resektion*	
Segmentektomie	173 (14 %)
Wedge-Resektion	115 (9,5 %)
Multiple Wedge-Resektionen	83 (7,6 %)
Bisegmentektomie	246 (20 %)
*Major-Resektion*	
HHR	243 (21 %)
HHL	119 (10 %)
Erweiterte HHR	51 (4,4 %)
Erweiterte HHL	30 (2,6 %)
*Kombinierte kolorektale Operation*	69 (8,4 %)
*Two-stage-Hepatektomie*	122 (11 %)
*Parenchymdissektoren*	
Andere	127 (10,5 %)
Thunderbeat®	111 (9,6 %)
Waterjet®	192 (17 %)
Harmonic Ace®	779 (67 %)
*Pringle-Manöver*	573 (49 %)
Dauer (min)	28 (15-45)
*Transfusion*	70 (6,1 %)
*Konversion*	49 (4,1 %)

^1^Kategorische Variablen werden als Häufigkeiten mit Prozentsätzen dargestellt (z. B. *n* [%]), nicht normalverteilte numerische Variablen werden durch den Median zusammen mit dem Interquartilsbereich dargestellt (z. B. Median [25.–75. Perzentil])

*HHR* Hemihepatektomie rechts, *HHL* Hemihepatektomie links

Eine Konversion auf ein offenes Verfahren fand in 49 Fällen (4,1 %) statt (Tab. 2). Die häufigsten Gründe waren Verwachsungen (39 %), komplexe Tumorlokalisation (14 %), gefolgt von der unklaren Gallengangsanatomie (12 %), der Nähe zu großen Gefäßen (10 %), einem großen tumorbedingten Leberlappen (8 %), einer Tumorperforation (4 %), CO_2_-Embolie (4 %) und Blutung (4 %), Tumorinfiltration anderer Organe (2 %) und einer komplexen Lymphadenektomie (2 %; Tab. 3). Die Konversionsrate zeigte keine signifikante Änderung über den Studienzeitraum (Zusatzmaterial online: Tab. 9).

**Tab. 3:** Aufgliederung der Konversionsgründe

Konversionsgründe	*N* = 49^1^
Verwachsungen	19 (38,8 %)
Tumorlokalisation	7 (14,3 %)
Gallengangsanatomie	6 (12,2 %)
Nähe zu großen Gefäßen	5 (10,2 %)
Großer Leberlappen	4 (8,1 %)
Tumorperforation	2 (4,1 %)
Blutungen	2 (4,1 %)
CO_2_-Embolie	2 (4,1 %)
Tumorinfiltration anderer Organe	1 (2 %)
Komplexe Lymphadenektomie	1 (2 %)

^1^ Kategorische Variablen werden als Häufigkeiten mit Prozentsätzen dargestellt (*n* [%])

In der univariaten Analyse zeigten 11 Faktoren eine Korrelation mit der Konversion (Zusatzmaterial online: Tab. 7). Nach Einschluss dieser Faktoren in die multivariate Analyse waren die Verwendung des Harmonic Ace® (Schätzer −1,25, 95 %-CI −1,99 bis −0,53) und des Waterjet® (Schätzer −1,70, 95 %-CI −3,19 bis −0,59) mit einer reduzierten Konversionsrate assoziiert (Zusatzmaterial online: Tab. 1 und 4). Mit einer erhöhten Konversionsrate waren die Hemihepatektomie links (Schätzer 0,99, 95 %-CI −0,015 bis 1,93) und der Durchmesser des größten Tumors (Schätzer 0,07, 95 %-CI 0,001 bis 0,148) assoziiert (Tab. 4).

**Tab. 4:** Multivariate Analyse von Faktoren und ihrer Assoziation mit der Konversion

Prädiktor	Schätzer	Standard-Error	Z‑Wert	*p*-Wert	Unteres CI (95 %)	Oberes CI (95 %)	Odds Ratio
(Interzept)	−3,99583902	0,69135372	−5,77973174	< 0,001	−5,468	−2,737	0,018
Harmonic Ace®	−1,22411128	0,36851848	−3,32170938	< 0,001	−1,962	−0,507	0,294
Waterjet®	−1,65204280	0,63907918	−2,58503617	0,01	−3,128	−0,539	0,192
HHL	1,15724344	0,46430891	2,49239982	0,013	0,195	2,036	3,181
Größter Tumordurchmesser	0,07450001	0,03468697	2,14778064	0,032	0,001	0,148	1,077
Vorherige Leberoperation	0,83771486	0,42934834	1,95113098	0,051	−0,029	1,668	2,311
Charlson-Komorbiditätsindex	0,15048858	0,08363692	1,79930802	0,072	−0,012	0,319	1,162
HCC	−1,66511433	1,19236357	−1,39648206	0,163	−4,738	0,302	0,189
IWATE-Score	0,079071959	0,08173385	0,96743221	0,333	−0,077	0,245	1,082
Neoadjuvante Chemotherapie	0,44967683	0,54408535	0,82648214	0,409	−0,582	1,558	1,568
Leberzirrhose	−0,45928743	1,19242594	−0,38517061	0,7	−3,531	1,513	0,632
CRLM	0,03132308	0,56976981	0,05497498	0,956	−1,040	1,206	1,032

*HHL* Hemihepatektomie links, *HCC* Hepatozelluläres Karzinom, *CRLM* Kolorektale Lebermetastasen

Die postoperative Major-Komplikationsrate (Clavien Dindo ≥ IIIa) betrug 18,3 % (Tab. 5). Der Comprehensive Complication Index lag bei einem Median von 0 (Interquartilsbereich 0–24). 11 % der Patienten hatten ein postoperatives Galleleck – davon 3,1 % ein Grad-A-, 7,4 % ein Grad-B- und 0,9 % ein Grad-C-Galleleck nach ISGLS. Bei 1,7 % der Fälle kam es zu einem postoperativen Leberversagen (PHLF). 1,2 % entwickelten ein Grad-A- 0,4 % ein Grad-B- und 0,2 % ein Grad-C-PHLF nach ISGLS. Der mediane postoperative Krankenhausaufenthalt betrug 6 Tage (Tab. 5).

**Tab. 5:** Postoperative Ergebnisse

Postoperatives Ergebnis	*N* = 12091
*Postoperativer Aufenthalt (Tage)*	6,0 (5,0–8,0)
*Clavien-Dindo-Klassifikation*	
0	735 (63 %)
I	80 (6,9 %)
II	139 (12 %)
IIIa	138 (12 %)
IIIb	44 (3,8 %)
IVa	12 (1,0 %)
IVb	4 (0,3 %)
V	14 (1,2 %)
*CCI*	0 (0–24)
*Postoperatives Leberversagen*	19 (1,7 %)
Grad A	13 (1,2 %)
Grad B	4 (0,4 %)
Grad C	2 (0,2 %)
*Postoperative Galleleckage *	125 (11 %)
Grad A	36 (3,1 %)
Grad B	85 (7,4 %)
Grad C	10 (0,9 %)

^1^ Kategorische Variablen werden als Häufigkeiten mit Prozentsätzen dargestellt (z. B. *n* [%]), nichtnormal verteilte numerische Variablen werden durch den Median zusammen mit dem Interquartilsbereich dargestellt (z. B. Median [25.–75. Perzentil]).

*CCI* Comprehensive Complication Index

**Tab. 6:** Multivariate Analyse von Faktoren und ihrer Assoziation mit Komplikationen

Prädiktor	Schätzer	Standard-Error	t‑Wert	*p*-Wert	Unteres CI (95 %)	Oberes CI (95 %)
HHR	9,399	1,828	5,141	< 0,001	5,811	12.988
Konversion	9,236	3,423	2,698	0,007	2,517	15.955
Alter	0,138	0,058	2,361	0,018	0,023	0,253
Neoadjuvante Chemotherapie	−3,647	2,314	−1,576	0,115	−8,188	0,895
CCC	4,536	3,187	1,423	0,155	−1,719	10.792
HCC	3,548	2,805	1,265	0,206	−1,957	9,054
CRLM	3,493	2,820	1,239	0,216	−2,041	9,027
Two-stage-Hepatektomie	2,902	2,349	1,236	0,217	−1,707	7,511
(Intercept)	7,801	8,615	0,905	0,365	−9,107	24.708
Bisegmentektomie	−1,626	1,819	−0,894	0,371	−5,196	1,943
Andere benigne Tumoren	2,421	3,239	0,747	0,455	−3,935	8,777
Robotisch	−5,407	7,321	−0,739	0,46	−19,775	8,960
Unilobär	−0,723	1,595	−0,454	0,65	−3,853	2,406

*HHR* Hemihepatektomie rechts, *CCC* Cholangiozelluläres Karzinom, *HCC* Hepatozelluläres Karzinom, *CRLM* Kolorektale Lebermetastasen

Nach univariater Analyse zeigten sich 11 Faktoren mit Komplikationen assoziiert (Zusatzmaterial online: Tab. 8). Diese Faktoren wurden in die multivariate Analyse eingeschlossen. Neben der Konversion (Schätzer 9,24, 95 %-CI 2,52 bis 15,96), zeigten die Hemihepatektomie rechts (Schätzer 9,4, 95 %-CI 5,81 bis 12,99) und das Alter (Schätzer 0,138, 95 %-CI 0,02 bis 0,25) der Patienten eine unabhängige Korrelation mit der Komplikationsrate (Tab. 6)

## Diskussion

Angesichts zunehmender Anwendung der minimal-invasiven Leberchirurgie dient diese Studie als wichtiger Leitfaden zur Stratifizierung und Identifikation von Patienten mit hohem Risiko für eine offene Konversion oder eine postoperative Komplikation. Unsere Ergebnisse bestätigen frühere Berichte, die eine ungünstigere Prognose für Patienten nach offener Konversion nahelegen [16]. Im Falle einer Konversion werden die Vorteile des minimal-invasiven Ansatzes teilweise oder ganz aufgehoben. Durch unsere systematische Analyse ist die präoperative Risikoabschätzung für Konversionen und Komplikationen möglich.

Im Studienzeitraum zeigte sich eine kontinuierliche Abnahme der offenen Resektionen von 77,7 % auf 23,6 %, während die Gesamtzahl der jährlichen Resektionen stabil blieb (Zusatzmaterial online: Tab. 9). Trotz zunehmender Komplexität der Eingriffe blieb die Konversionsrate konstant (Zusatzmaterial online: Tab. 9). Mit einer durchschnittlichen Rate von 4,1 % war diese im internationalen Vergleich eher niedrig ([16, 18]; Tab. 2). Wir führen dies auf eine schrittweise Zunahme minimal-invasiver Operationen, wachsende operative Erfahrung und damit verbundene Lerneffekte zurück.

Intraoperative Blutungen sind laut Literatur ein häufiger Grund für Konversionen [18]. In unserer Kohorte war dies lediglich in zwei Fällen ursächlich (Tab. 3). Wir gehen davon aus, dass die intraoperativen Blutungen mit zunehmender Erfahrung minimal-invasiv besser beherrscht werden können [13]. Diese Annahme wird durch die vergleichsweise niedrige Transfusionsrate von 6,1 % gestützt (Tab. 2).

Unsere Analyse identifizierte mehrere unabhängige Prädiktoren für Konversionen. Signifikant waren die linksseitige Hemihepatektomie sowie große Tumoren (bezogen auf den maximalen Durchmesser). Hinsichtlich der verwendeten Dissektionsinstrumente war der Einsatz von Harmonic Ace® und Waterjet® mit einer geringeren Konversionsrate assoziiert (Tab. 4). Technische Schwierigkeiten, wie Verwachsungen, eingeschränkte Exposition, erschwerte Mobilisation oder das Erreichen sicherer Resektionsränder, stellen die häufigsten Gründe für eine Konversion dar. Weitere Ursachen waren eine ungünstige Tumorlokalisation, komplexe Gallengangsanatomien, die Nähe zu großen Gefäßen sowie ein (tumorbedingter) voluminöser und somit nicht mobilisierbarer Leberlappen. In zwei Fällen kam es zu einer intraoperativen Tumorperforation, in einem Fall lag eine Mageninfiltration vor, und in einem weiteren war eine komplexe Lymphadenektomie mit Konversion notwendig (Tab. 3). Der chirurgische Zugang (laparoskopisch oder robotisch) war interessanterweise kein signifikanter Prädiktor für eine Konversion (s. Zusatzmaterial online: Tab. 7).

Seit der Einführung robotergestützter Systeme wurden die technische Limitation der Laparoskopie – insbesondere in posterosuperioren Segmenten – teilweise überwunden [14]. Es gibt Hinweise auf eine kürzere Lernphase im Vergleich zur laparoskopischen Leberchirurgie [13]. Dennoch ist die Evidenzlage für eine Überlegenheit robotischer Verfahren weiterhin heterogen und vorwiegend durch retrospektive Daten geprägt. Bisherige prospektive randomisierte Studien sind auf Basis der zu erwartenden Effektstärken unzureichend gepowert [3].

Der IWATE-Score hat sich zur Abschätzung des operativen Schwierigkeitsgrades bei minimal-invasiven Leberresektionen etabliert. Trotz Ähnlichkeiten zwischen der laparoskopischen und robotischen Leberchirurgie ist bisher unklar, inwieweit sich der IWATE-Score auf den robotischen Ansatz übertragen lässt. Insbesondere der Schwierigkeitsgrad der posterosuperioren Segmente und der Faktor „Handassistenz“ sollten bei einer Überarbeitung des IWATE-Scores für robotische Leberresektionen angepasst werden.

Im Vergleich zur deutschlandweiten Morbidität von 24,3 % und einer Mortalität von 7,7 % [2] bzw. 4,6 % in High-volume-Zentren [9] für Leberchirurgie konnte mit der minimal-invasiven Technik an unserem Zentrum eine niedrigere 90-Tages-Morbidität (18,6 %) und Mortalität (1,2 %) erreicht werden (Tab. 4). Diese Zahlen unterstreichen die Bedeutung der minimal-invasiven Leberchirurgie als komplikationsarmes Verfahren, das bei entsprechender Patientenselektion und Expertise einen Beitrag zu guten postoperativen Ergebnissen liefern kann.

Diese Studie hat wichtige klinische Implikationen: Sie kann die Patientenselektion für die minimal-invasive Leberchirurgie verbessern, die Wahl des Zugangsweges und der Dissektionsinstrumente erleichtern und damit das Risiko ungeplanter Konversionen sowie potenzieller Komplikationen senken.

## Limationen

Durch das retrospektive Studiendesign ergeben sich Einschränkungen durch Selektionsverzerrungen und unkontrollierte Confounder. Aussagen zur Kausalität können nicht getroffen werden. Bei einem Vergleich zwischen laparoskopischem und robotischem Zugang ist aufgrund einer nicht klar definierten Indikationsstellung eine Selektionsverzerrung möglich.

## Ausblick

Zusammenfassend hebt diese Studie die Faktoren hervor, die mit einer offenen Konversion bei minimal-invasiven Leberresektionen verbunden sind. Die offene Konversion war ein unabhängiger Prädiktor für eine erhöhte Komplikationsrate.

## Fazit für die Praxis

- Die minimal-invasive Leberchirurgie zeigt eine niedrige Morbidität und Mortalität.
- Konversionen sind mit einer erhöhten Komplikationsrate assoziiert.
- Technische Gründe und Verwachsungen sind die häufigsten Ursachen für eine Konversion.
- Die linksseitige Hemihepatektomie und große Tumoren sind mit einer erhöhten Konversionsrate assoziiert.

## Supplementary Information

Tab. 7 Univariate Analyse von Faktoren und ihrer Assoziation mit der Konversion

Tab. 8 Univariate Analyse von Faktoren und ihrer Assoziation mit Komplikationen

Tab. 9 Jährliche Eingriffszahlen mit Konversionsraten und Schwierigkeitsgrad

## Data Availability

Die erhobenen Datensätze können auf begründete Anfrage in anonymisierter Form beim korrespondierenden Autor angefordert werden. Die Daten befinden sich auf einem Datenspeicher an der Chirurgischen Klinik | CCM | CVK der Charité.

## Abkürzungen

ASA: American Society of Anesthesiologists
CRLM: Kolorektale Lebermetastasen
HCC: Hepatozelluläres Karzinom
PHBL: Postoperative Galleleckage
PHLF: Posthepatektomieleberversagen
