# Supplementary material for: Influencing factors and results of conversions in minimally invasive liver surgery: A single-center analysis of over 1200 consecutive cases
Source: Chirurgie (Heidelb). 2025 Sep 11;97(5):397–404. [Article in German] doi: 10.1007/s00104-025-02374-0 (PMC13106248; doi:10.1007/s00104-025-02374-0)
Supplement: Supplementary file 1 — Tab. 7 Univariate Analyse von Faktoren und ihrer Assoziation mit der Konversion [file 104_2025_2374_MOESM1_ESM.pdf]

Tabelle 7: Univariate Analyse von Faktoren und ihrer Assoziation mit der Konversion

Prädiktor	Schätzer	Standard Error	Z-Wert	P-Wert	Unteres CI (95%)	Oberes CI (95%)	Odds Ratio
Harmonic Ace	-0.9992608276	0.286608140	-3.48650540	<0.001	-1.561	-0.438	0.368
Neoadjuvante Chemotherapie	1.0441241033	0.316266166	3.30140943	<0.001	0.424	1.664	2.841
IWATE Schwierigkeitsgrad	0.1764683120	0.058014024	3.04182160	0.002	0.063	0.290	1.193
CRLM	0.8393816374	0.285556833	2.93945562	0.003	0.280	1.399	2.315
HHL	1.0012484858	0.343619546	2.91382867	0.004	0.328	1.675	2.722
HCC	-1.6288559983	0.598626972	-2.72098665	0.007	-2.802	-0.456	0.196
Vorherige Leberoperation	0.8716805671	0.325120910	2.68109660	0.007	0.234	1.509	2.391
Größter Tumordurchmesser	0.0421061483	0.015822768	2.66111133	0.008	0.011	0.073	1.043
Leberzirrhose	-1.6159953130	0.725435242	-2.22762174	0.026	-3.038	-0.194	0.199
Charlson Comorbiditätsindex	0.1189181458	0.054422621	2.18508669	0.029	0.012	0.226	1.126
Waterjet	-1.2096334264	0.599959807	-2.01619077	0.044	-2.386	-0.034	0.298
Minor Resektion	-0.5455547582	0.284731545	-1.91603202	0.055	-1.104	0.013	0.580
Cholangiokarzinom	0.7255703673	0.380563373	1.90656910	0.057	-0.020	1.471	2.066
Nähe zu großen Gefäßen	0.5377528742	0.284855140	1.88781173	0.059	-0.021	1.096	1.712
Bilobäre Lokalisation	-0.4590745587	0.281827332	-1.62892135	0.103	-1.011	0.093	0.632
Two-Stage Resektion	0.5581304227	0.380946363	1.46511551	0.143	-0.189	1.305	1.747
Andere gutartige Tumoren	-0.6206244361	0.440171946	-1.40995909	0.159	-1.483	0.242	0.538
Erw. HHL	0.8782426246	0.625530259	1.40399703	0.16	-0.348	2.104	2.407
Thunderbeat	-1.0074809373	0.728046488	-1.38381402	0.166	-2.434	0.419	0.365
Body mass index	0.0318355952	0.028140366	1.13131417	0.258	-0.023	0.087	1.032
Segmentektomie	-0.5377218033	0.477332320	-1.12651455	0.26	-1.473	0.398	0.584
Wedge Resektion	-0.6261563116	0.602709681	-1.03890203	0.299	-1.807	0.555	0.535
Robotisch	0.3125886117	0.302195629	1.03439157	0.301	-0.280	0.905	1.367
Laparoscopisch	-0.2705289932	0.302039991	-0.89567276	0.37	-0.863	0.321	0.763
Steatose	-0.0076468411	0.009168374	-0.83404551	0.404	-0.026	0.010	0.992
ASA Score	0.8405306571	1.034612959	0.81241072	0.417	-1.187	2.868	2.318
Anderer Maligne Tumore	-0.3669692077	0.529114227	-0.69355385	0.488	-1.404	0.670	0.693
Geschlecht	0.1968496574	0.291569482	0.67513807	0.5	-0.375	0.768	1.218
Bisegmentektomie	-0.1493663456	0.358585585	-0.41654308	0.677	-0.852	0.553	0.861
Kombinierte Kolorektale Operation	0.1648446988	0.542043884	0.30411689	0.761	-0.898	1.227	1.179
Erw. HHR	-0.1644743898	0.735284192	-0.22368819	0.823	-1.606	1.277	0.848
Multiple Wedge Resektionen	0.0517994835	0.534703434	0.09687517	0.923	-0.996	1.100	1.053

Prädiktor	Schätzer	Standard Error	Z-Wert	P-Wert	Unteres CI (95%)	Oberes CI (95%)	Odds Ratio
HHR	-0.0180184993	0.346629827	-0.05198196	0.959	-0.697	0.661	0.982
Pringle Manöver	-0.0142523418	0.281239966	-0.05067680	0.96	-0.565	0.537	0.986
Alter	0.0003357432	0.010179526	0.03298220	0.974	-0.020	0.020	1.000
Hybrid	-13.5323861993	799.848252147	-0.01691869	0.987	-1,581.206	1,554.141	0.000
