# Supplementary material for: Influencing factors and results of conversions in minimally invasive liver surgery: A single-center analysis of over 1200 consecutive cases
Source: Chirurgie (Heidelb). 2025 Sep 11;97(5):397–404. [Article in German] doi: 10.1007/s00104-025-02374-0 (PMC13106248; doi:10.1007/s00104-025-02374-0)
Supplement: Supplementary file 2 — Tab. 8 Univariate Analyse von Faktoren und ihrer Assoziation mit Komplikationen [file 104_2025_2374_MOESM2_ESM.pdf]

Tabelle 8: Univariate Analyse von Faktoren und ihrer Assoziation mit Komplikationen

Prädiktor	Schätzer	Standard Error	t-Wert	P-Wert	Unteres CI (95%)	Oberes CI (95%)
HHR	11.737	1.935	6.065	<0.001	7.940	15.535
Konversion	13.802	3.792	3.640	<0.001	6.363	21.241
Unilobär	-5.556	1.679	-3.309	<0.001	-8.851	-2.261
Neoadjuvante Chemotherapie	-5.345	1.672	-3.197	0.001	-8.626	-2.064
HCC	5.869	1.990	2.949	0.003	1.964	9.773
CRLM	-4.884	1.707	-2.861	0.004	-8.233	-1.534
Two-Stage Hepatektomie	6.016	2.161	2.784	0.005	1.775	10.257
Robotisch	5.087	1.898	2.680	0.007	1.362	8.811
Alter	0.144	0.060	2.403	0.016	0.026	0.262
Bisegmentektomie	-4.915	2.053	-2.394	0.017	-8.943	-0.887
CCC	6.727	2.825	2.382	0.017	1.185	12.269
Andere benigne Tumore	-4.455	2.129	-2.093	0.037	-8.631	-0.278
Voroperation Leber	4.210	2.195	1.918	0.055	-0.096	8.517
ASA Score	6.994	3.763	1.859	0.063	-0.389	14.377
Segmentektomie	-4.128	2.359	-1.750	0.08	-8.756	0.499
Geschlecht	2.687	1.701	1.580	0.114	-0.650	6.024
BMI	0.225	0.163	1.380	0.168	-0.095	0.544
Wedge Resektion	-3.821	2.807	-1.361	0.174	-9.329	1.687
Charlson Komorbiditätsindex	-0.414	0.305	-1.355	0.176	-1.013	0.185
Erw. HHR	5.184	3.867	1.341	0.18	-2.402	12.770
HHL	-2.438	2.630	-0.927	0.354	-7.599	2.723
Andere maligne Tumore	2.555	2.766	0.924	0.356	-2.872	7.982
Maximale Tumorgröße	0.132	0.149	0.888	0.375	-0.160	0.425
Leber Zirrhose	-1.872	2.184	-0.857	0.392	-6.158	2.414
Erw. HHL	-3.999	5.015	-0.797	0.425	-13.839	5.841
Nähe zu großen Gefäßen	1.100	1.602	0.687	0.492	-2.043	4.243
Pringle Manöver	0.025	0.037	0.674	0.5	-0.047	0.096
Hybrid Operation	6.291	9.573	0.657	0.511	-12.492	25.074
Steatose Leber	1.033	1.679	0.616	0.538	-2.261	4.328
Harmonic Ace	-0.671	1.697	-0.395	0.693	-3.999	2.658
Kombinierte kolorekteale Operation	0.310	3.703	0.084	0.933	-6.959	7.579
Multiple Wedge Resektionen	-0.242	3.370	-0.072	0.943	-6.854	6.370
Waterjet	-0.099	2.139	-0.046	0.963	-4.296	4.099
Thunderbeat	-0.104	2.702	-0.038	0.969	-5.405	5.197
