# Supplementary material for: Influencing factors and results of conversions in minimally invasive liver surgery: A single-center analysis of over 1200 consecutive cases
Source: Chirurgie (Heidelb). 2025 Sep 11;97(5):397–404. [Article in German] doi: 10.1007/s00104-025-02374-0 (PMC13106248; doi:10.1007/s00104-025-02374-0)
Supplement: Supplementary file 3 — Tab. 9 Jährliche Eingriffszahlen mit Konversionsraten und Schwierigkeitsgrad [file 104_2025_2374_MOESM3_ESM.pdf]

**Tabelle 9:** Jährliche Eingriffszahlen mit Konversionsraten und Schwierigkeitsgrad

| Jahr | 2015 | 2016 | 2017 | 2018 | 2019 | 2020 | 2021 | 2022 | 2023 | 2024 | p-Wert |
|---|---|---|---|---|---|---|---|---|---|---|---|
| **Gesamt** | 224 | 256 | 262 | 308 | 269 | 250 | 239 | 246 | 263 | 161 | |
| **Offene Operationen** | 174 (77,7%) | 179 (69,9%) | 165 (63%) | 180 (58,4%) | 131 (48,7%) | 115 (46%) | 83 (34,7%) | 112 (45,5%) | 92 (35%) | 38 (23,6%) | <0,001[a] |
| **Minimalinvasiv** | 50 | 77 | 97 | 128 | 138 | 135 | 156 | 134 | 171 | 123 | |
| **Konversionen** | 0 (0%) | 6 (6,5%) | 2 (2,1%) | 3 (2,3%) | 2 (1,4%) | 10 (7,4%) | 12 (7,7%) | 8 (6%) | 9 (5,3%) | 2 (1,6%) | 0,26[a] |
| **IWATE-Score** | 6 (4-9) | 6,5 (5-9) | 7 (5-9) | 6 (5-9) | 8 (5-10) | 8 (5-10) | 8 (5-10) | 8 (5,75-10) | 9 (5-10) | 7 (5-10) | <0,001[b] |

Kategorische Variablen werden als Häufigkeiten mit Prozentsätzen dargestellt. n (%), nicht normal verteilte numerische Variablen werden durch den Median zusammen mit dem Interquartilsbereich dargestellt (z. B. Median [25. - 75. Perzentil]). [a] Cochran-Armitage trend test, [b] Jonckheere-Terpstra Trend Test
